# Potential Satellite Cell-Linked Biomarkers in Aging Skeletal Muscle Tissue: Proteomics and Proteogenomics to Monitor Sarcopenia

**DOI:** 10.3390/proteomes10030029

**Published:** 2022-08-19

**Authors:** Diego Fernández-Lázaro, Evelina Garrosa, Jesús Seco-Calvo, Manuel Garrosa

**Affiliations:** 1 Department of Cellular Biology, Genetics, Histology and Pharmacology, Faculty of Health Sciences, University of Valladolid, Campus of Soria, 42003 Soria, Spain; 2 Neurobiology Research Group, Faculty of Medicine, University of Valladolid, 47005 Valladolid, Spain; 3 Department of Cell Biology, Genetics, Histology and Pharmacology, Faculty of Medicine, Institute of Neurosciences of Castile and Leon (INCYL), University of Valladolid, 47005 Valladolid, Spain; 4 Institute of Biomedicine (IBIOMED), Physiotherapy Department, University of Leon, Campus de Vegazana, 24071 Leon, Spain; 5 Psychology Department, Faculty of Medicine, Basque Country University, 48900 Leioa, Spain

**Keywords:** sarcopenia, proteogenomic, proteomics, liquid biopsy, tissue biopsy, muscle satellite cells, aging, biomarkers

## Abstract

Sarcopenia (Sp) is the loss of skeletal muscle mass associated with aging which causes an involution of muscle function and strength. Satellite cells (Sc) are myogenic stem cells, which are activated by injury or stress, and repair muscle tissue. With advancing age, there is a decrease in the efficiency of the regenerative response of Sc. Diagnosis occurs with the Sp established by direct assessments of muscle. However, the detection of biomarkers in real-time biofluids by liquid biopsy could represent a step-change in the understanding of the molecular biology and heterogeneity of Sp. A total of 13 potential proteogenomic biomarkers of Sp by their physiological and biological interaction with Sc have been previously described in the literature. Increases in the expression of GDF11, PGC-1α, Sirt1, Pax7, Pax3, Myf5, MyoD, CD34, MyoG, and activation of Notch signaling stimulate Sc activity and proliferation, which could modulate and delay Sp progression. On the contrary, intensified expression of GDF8, p16INK4a, Mrf4, and activation of the Wnt pathway would contribute to early Sp development by directly inducing reduced and/or altered Sc function, which would attenuate the restorative capacity of skeletal muscle. Additionally, tissue biopsy remains an important diagnostic tool. Proteomic profiling of aged muscle tissues has shown shifts toward protein isoforms characteristic of a fast-to-slow transition process and an elevated number of oxidized proteins. In addition, a strong association between age and plasma values of growth differentiation factor 15 (GDF-15) has been described and serpin family A member 3 (serpin A3n) was more secreted by atrophied muscle cells. The identification of these new biomarkers holds the potential to change personalized medicine because it could predict in real time the course of Sp by monitoring its evolution and assessing responses to potential therapeutic strategies.

## 1. Introduction

The organism changes with age due to transformations that occur in individual cells and in the organs. These physiological changes lead to changes in internal functions and to a drastic decrease in the repair and regenerative potential of the tissues [1]. One of the most relevant changes of aging, due to its dramatic consequences, is the loss of skeletal muscle mass, strength, and muscle motor function [2]. Additionally, the state of sarcopenia is a decrease in the functionality of satellite cells (Sc), which modifies the capacity for muscle restoration in old age [2]. This process of skeletal muscle involution was described by Rosenberg, calling it “*Sarcopenia*” (from the Greek *sarx*, flesh, and *penia*, poverty), which also includes the qualitative changes in muscle tissue associated with aging [3]. Sarcopenia, according to the *European Working Group on Sarcopenia in Older People* (EWGSOP) defines it as a progressive and generalized skeletal muscle disorder, which is associated with an increased likelihood of adverse effects such as falls, fractures, disability, and mortality. According to losses in muscle strength, physical performance, and quantity/quality of skeletal muscle mass, EWGSOP established three grades: Presarcopenia, Sarcopenia, and Severe Sarcopenia [4].

Sarcopenia is a considerable public health problem due to the progressive aging of the world population. The increase in life expectancy together with a reduced birth rate causes the population pyramid to invert and the number of cases to increase [5]. The prevalence increases by 5–13% between 60 and 70 years of age, and by 11–50% among those over 80 years of age [6]. In 2050, the population over 60 years of age will double and 30% of the population will be over 60 years [6]; thus, the number of patients afflicted by sarcopenia will be dramatically increased.

## 2. Factors That Contribute to the Development of Sarcopenia

**Figure 1:**
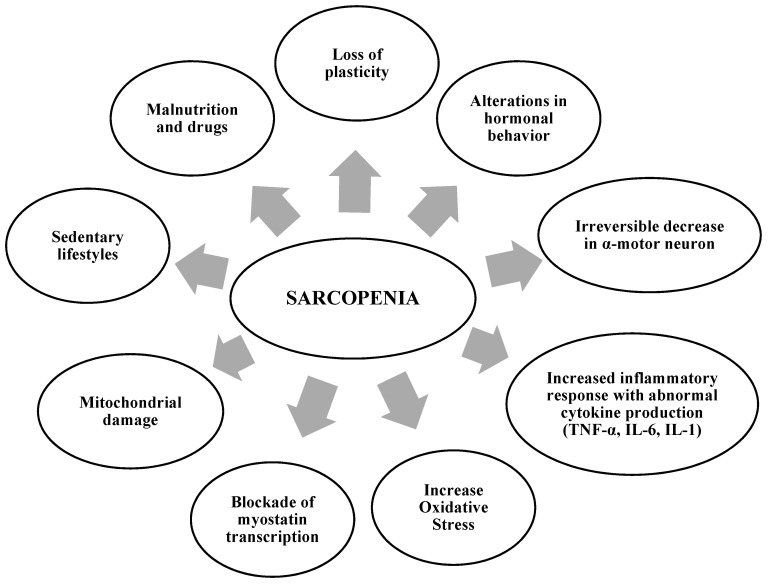
Main factors associated with the development of sarcopenia etiopathogenesis.

There are several biological processes that influence the origin and evolution of sarcopenia; therefore, the etiopathogenesis of sarcopenia is multifactorial (Figure 1). In addition, a sedentary lifestyle, deficient protein nutrition, and certain drugs are responsible for triggering sarcopenic processes [5].

Loss of plasticity reduces the muscle’s ability to adapt to functional demands, affecting neuromuscular activity, hormonal metabolism, and the transformation of different types of muscle fibers [7]. Probably the loss of plasticity derives from the infiltration of fat in the muscle tissue, both among the muscle groups and lipid droplets inside the skeletal myocytes, and then the processes of massive infiltration of adipocytes would trigger sarcopenic obesity. Changes in hormonal status occur in both the production and in the sensitivity of growth hormone (GH), insulin-like growth factor I (IGF-1), corticosteroids, androgens, estrogens, and insulin. Alterations in hormonal behavior in sarcopenia stimulate catabolic processes resulting in increased visceral fat and decreased muscle mass [8]. The changes in the nervous system caused by aging lead to a progressive and irreversible decrease in motor units (α-motor neurons). The loss of α-motor neurons, segmental demyelination, and the lack of conduction of the nerve impulse to type II muscle fibers simultaneously with their selective denervation, causes alterations in contractile capacity with decreased movement execution [9]. Previously, it has been determined that sarcopenia and associated pathologies present a chronic inflammatory response characterized by abnormal cytokine production (tumor necrosis alfa (TNF-α), interleukins 6 (IL-6) and 1 (IL-1)), increased acute phase reactants (C-reactive protein (CRP)), and trigger inflammatory signaling pathways, most commonly the NF-κB, MAPK, and JAK-STAT pathways [10]. TNF-α plasma increase activates the ubiquitin–protease pathway that stimulates proteolysis [11]. Taken together, the inflammatory response triggers a catabolic and anorexigenic effect [9]. Increased oxidative stress in aging generates an imbalance between oxidative agents and the myocyte antioxidant response. This imbalance results in increased catabolic signaling and impaired anabolic signaling. The accumulation of reactive oxygen species (ROS) causes mitochondrial DNA (mtDNA) damage, downregulation of protein synthesis and myocyte adenosine triphosphate (ATP) production capacity, and stimulation of muscle senescence processes. In addition, excess of ROS downregulates Nrf2 expression, which blocks the response of the “*Antioxidant Response Element*” system and activates apoptotic pathways (intrinsic and extrinsic) causing excessive cell death and impairing regenerative capacity in skeletal muscle [12]. Aging activates the blockade of myostatin transcription leading to muscle atrophy of fast fibers and limitations of movement in the organism. In addition, the increase in angiotensin-converting enzyme (ACE) activity produces higher levels of angiotensin II and lower levels of bradykinin, which stimulate a degenerative action on muscle tissue. Certain polymorphisms in the vitamin D receptor gene influence striated muscle and some of these have been associated with the appearance of sarcopenia in the elderly [1,13,14].

Sedentary lifestyles or low levels of physical activity are linked to a rapid loss of muscle mass and strength. These muscle alterations lead to: difficulties in walking and balance, leading to a greater sedentary lifestyle; an increased risk of falls, associated with an increased risk of fractures; and a deterioration in the quality of life [13]. Additionally, the risk of sarcopenic obesity is increased leading to lipid toxicity and an additional inflammatory state [11]. The decrease in food intake and the lack of essential nutrients such as proteins lead to significant levels of protein malnutrition resulting in anorexia in aging. The anorexia of aging, which is characterized by a lack of appetite, shows slowing of gastric emptying causing several comorbidities that accentuate notably the muscular wasting and functional deterioration [15]. Older adults are prescribed chronic use of drugs such as glucocorticoids, beta-blockers, hydroxy-methyl-glutaryl-COA reductase inhibitors, or non-steroidal anti-inflammatory drugs which cause side effects on muscle such as mitochondrial alterations and muscle atrophy, among other toxicities [16].

## 3. Biological Basis of Skeletal Muscle in Aging

Skeletal muscle is mainly composed of two main fiber types: (a) type I myofibers, which have a slow contraction time, use oxidative pathways, and resist fatigue; and (b) type II myofibers, which have a fast contraction time, rely on glycolytic pathways, and promote fatigue more readily. This structural composition of muscle fibers is affected by various mechanisms that are related to increasing age [17]. Type II fibers generally tend to progressively decrease with the aging process; on the contrary, type I fibers do not present any change and increase their number in compensation, producing an imbalance that is evidenced in the decrease in muscular oxidative activity and capillary density [13]. The morpho-functional unit of the muscle “*sarcomere*” presents a degenerative process that occurs through the replacement of the muscle fiber with fibrous tissue and fat, which leads to a shortening of the fibers that compose the muscle reducing its contraction capacity [18]. Additionally, there is evidence of a decrease at the central level of the α motor neurons of the spinal ventral gray horn. This loss of α-motor neurons leads to the recruitment of the denervated myocytes by the existing motor units but reaches a muscular work overload that is hardly assumable by the sarcopenic muscle [19].

**Figure 2:**
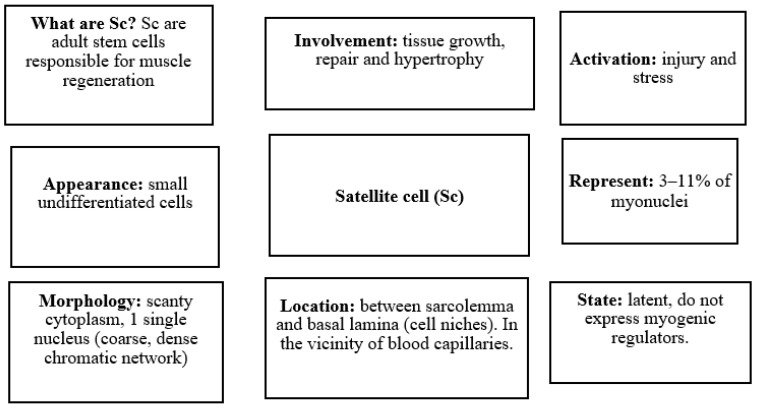
What are the characteristics that differentiate satellite cells?

Sc are a group of cells in muscle structures that are undifferentiated, i.e., they lack tissue specialization and can differentiate into myoblasts (Figure 2). These distinct cells were discovered by the biophysicist Alexander Mauro more than 50 years ago (1961) in the periphery of human skeletal muscle fibers [20]. Although initially defined by this distinct anatomical location, adult Sc remain quiescent in the sublaminar niche adjacent to the myofiber. Sc show scant cytoplasm and a coarse chromatic nucleus [21]. Sc are responsible for muscle growth and repair in response to stimuli that trigger the progression of Sc through the myogenic program that is tightly regulated by a hierarchy of pathways [22]. Sc remain dormant until the muscle fiber is damaged, at which time they are activated and begin to express myogenic regulators to give rise to new myoblasts. These myoblasts fuse within the outer basal lamina to form myotubes (elongated multinucleated elements) that increase in volume until the muscle fiber is rebuilt. Conversely, if the outer basal lamina is destroyed, fibroblasts repair the injured tissue [7,21]. Sc can exist in two different states: quiescent and activated. Histologically, quiescent Sc are undifferentiated mononucleated cells, with few organelles, a small nucleus relative to the adjacent myotube, and more transcriptionally inactive heterochromatin as compared to the muscle fiber nucleus [23]. Quiescent Sc is in a state of reversible cell cycle arrest and can be activated following proliferative pressure imposed by the homeostatic demand of a renewing tissue, or regenerative demand following injury or damage. After activation, these stem cells re-enter the cell cycle and proliferate [24]. Active Sc are observed as a bulge in the myofiber. This increase in mitotic activity induces an increase in the number of caveolae, a reduction in the amount of heterochromatin, an increase in the number of organelles, and an increase in the cytoplasmic-to-nuclear ratio [21,22]. The activated Sc automatically coordinate the renewal of a new stem cell guaranteeing the maintenance of Sc and their differentiation to create specialized mature progeny that can replace the lost muscle cells [24].

The amounts of Sc in adult muscle can vary between 3 and 11% of myonuclei; however, older adults own 40% less, suggesting that with age there is a decline in the population of the Sc [12]. Sarcopenia is also accompanied by a significant decrease in Sc function, which strongly compromises the regenerative capacity of skeletal muscle. Sc loss contributes to muscle fibrosis in old age and decreases the ability of sarcopenic muscles to recover in response to injury [25]. Thus, Sc may be the major target of the sarcopenic aging process. Sarcopenia compromises Sc maintenance and function, which, in turn, contributes to diminished regenerative capacity and tissue homeostasis throughout life. This link is the basis for the postulation of a stem-cell-centered theory of sarcopenic aging [26].

## 4. Liquid Biopsy as a New Tool in Precision Medicine: Potential Application in Sarcopenia

The set of Sc activities such as quiescence, activation, self-renewal, and differentiation are coordinated through the interaction between intrinsic programs and signals from the surrounding environment, termed the “*niche*”. The Sc niche, composed of supporting cell types, local growth factors, and extracellular matrix molecules, is crucial for directing stem cell self-renewal and differentiation during muscle regeneration [23]. Therefore, the Sc aging process would be a consequence of the combined effects of age-dependent alterations in the environment and intrinsic dysregulations associated with the Sc [27]. In this scenario, the recognition of the mechanisms responsible for the evolution of sarcopenia remains a difficult task. However, in recent years there has been a remarkable increase in the knowledge of aging, accompanied by a very important technological development of high-sensitivity molecular biology techniques that introduces us to the beginning of the application of precision medicine [28]. Precision medicine in the clinical management of some diseases, including sarcopenia, can be achieved through the diagnostic platform called “*liquid biopsy*” (LB). LB was recognized as a powerful real-time approach for molecular monitoring of diseases, such as cancer [29]. This method uses biomarker detection for prognostic and predictive purposes by noninvasive means, which soon will represent a paradigm shift in the understanding of molecular biology and disease heterogeneity [28,29].

**Figure 3:**
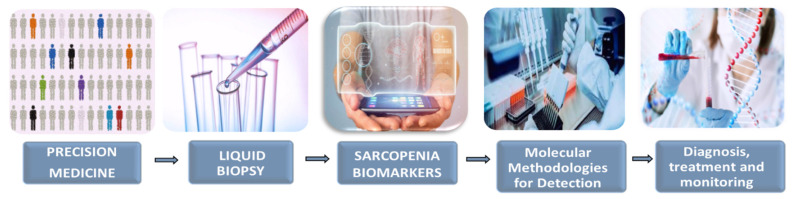
The operational flow of liquid biopsy: an individualized strategy to capture the dynamics of sarcopenia.

Currently, methods for diagnosing sarcopenia are effective when the disease is already established, through measuring muscle mass or mobility loss, muscle biopsies, and imaging [30]. The outcome of these diagnoses may be altered by the comorbidities of the sarcopenia population [4]. Some clinical applications of LB in oncology, which could be applicable to aging, have been suggested, such as molecular profiling, diagnosis, treatment response monitoring, early detection, screening, and determination of triggering pathways [28]. Therefore, it is essential to specify biomarkers in sarcopenia, which will allow for its understanding and serve as tools to guide diagnosis, treatment, and monitoring [30]. Biomarkers are understood as molecules found in different biofluids that indicate a physiological or pathological change [28]. However, technological, instrumental, and scientific difficulties pose a challenge for LB until it is used in the clinic in a standardized way. Therefore, it is mandatory to previously validate and establish a biomarker panel that establishes standardized working protocols for real-time monitoring of sarcopenia. Additionally, it is necessary to evaluate whether the biomarkers found in biofluids reproduce the results of tissue biopsies [28,29] (Figure 3).

LB is the use of patients’ body fluids present in non-solid biological tissue such as blood, urine, saliva, urine, cerebrospinal fluid, and pleural effusion. The biomarkers are Sc, cell-free circulating nucleic acids, exosomes, and platelets. Sc are difficult to isolate given their heterogeneous morphology and low numbers in circulation [28]. There are still no screening tests for Sc by LB in sarcopenic elderly; however, in patients with metastatic cancer, there are between 5 and 50 circulating tumor cells in 7.5 mL of blood. In patients with earlier cancer stages, only 10 circulating tumor cells are detected on average in 7.5 mL of blood. Therefore, it is the circulating nucleic acids that would take on greater prominence and importance in the field of LB [29]. In the monitoring of cancer patients, nucleic acids are presented as a component of the liquid biopsy capable of providing detailed information about tumor heterogeneity. As circulating tumor DNA can originate from any tumor cell, each with presumably different genomic alterations, it is possible to detect a large spectrum of tumor mutations. In this regard, the average amount of circulating tumor DNA in 1 mL of plasma from advanced-stage cancer patients (17 ng) and the amount of DNA contained by a single human cell (6 pg), would require more than 2000 circulating tumor cells to release their genetic content into that volume of plasma [28,29]. In previous studies, different molecular detection technologies employed in WL are described: quantitative polymerase chain reaction quantitative polymerase chain reaction (q-PCR), BEAMing, safe sequencing system (Safe-SeqS), personalized cancer profiling by deep sequencing (CAPP-Seq), digital polymerase chain reaction (dPCR), copy number aberrations (CNA) or point mutations by whole-genome sequencing (whole genome sequencing, WGS) or whole-exome sequencing (whole-exome sequencing, WES) and tagged amplicon deep sequencing (TAmSeq) [28].

## 5. Potential Proteogenomic Biomarkers Involved in Sarcopenia

**Figure 4:**
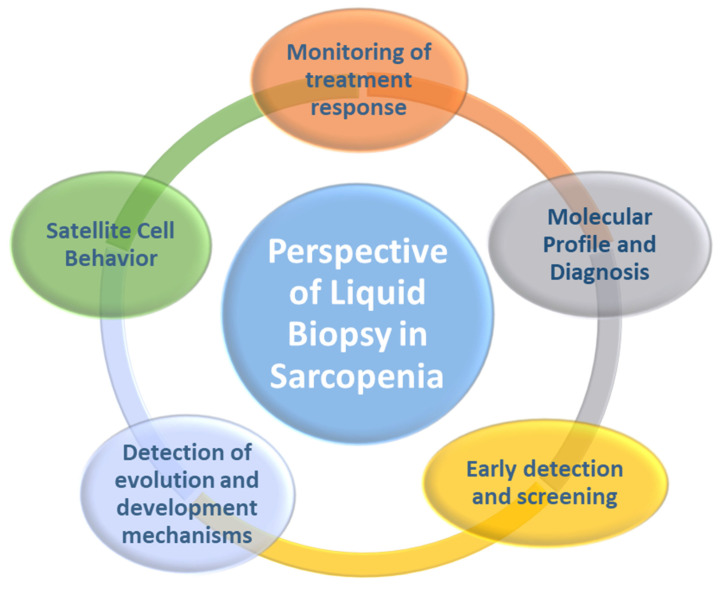
Potential applications of liquid biopsy in the management of sarcopenia.

We considered different biomarkers biologically and/or functionally linked to Sc that could be monitored in the biofluids of sarcopenic patients, and thus be able to choose the right approach to improve the health of older adults. This could transform the management of Sp because it would facilitate tracking treatment response, molecular profiling and diagnosis, early detection and screening, mechanisms of progression and development, and monitoring of the behavior of Sc (Figure 4). Some advantages of biomarker determination by LB over tissue biopsy would be minimally invasive, easy to obtain from the patient’s blood, less expensive, a short processing time, low failure rate, serial biopsies can be tolerated throughout the aging process, the sample can remain stable for long periods of time in ex vivo conditions, it can capture the heterogeneity of skeletal muscle, and responses to interventions are easy to follow [28].

### 5.1. Notch Signaling

Previous evidence shows the important role of Notch in myogenesis [31]. Notch is a master regulator of Sc function and its balance controls Sc self-renewal and myogenic differentiation in a coordinated manner [32]. The delta (Δ) ligand interacts with the Notch receptor, which is cleaved and translocated into the nucleus, where it upregulates transcription factors (Hes1, Hey1), leading to myoblast proliferation. Insufficient Notch activation contributes to the diminished regenerative capacity of aged muscle and loss of Notch function may be at least part of the mechanism leading to depletion in the Sc pool [33]. Indeed, aged Sc fail to adequately regulate the Notch Δ ligand following muscle injury and show consistent defects in cell cycle entry and exit [34]. In animal models, decreased Notch expression in activated aged Sc corresponds with decreased myoblast proliferation and young Sc, as well as Notch activation in aged muscle increased Sc activation and myoblast proliferation [31]. Notch signaling synergizes with Wnt signaling in Sc to control muscle regeneration [34].

Additionally, muscle aging may be associated with a decrease in upstream factors that activate Notch signaling, such as mitogen-activated protein kinase (MAPK) and phosphate extracellular signal-regulated kinase (pERK). MAPK/pERK is depressed in aged muscle, which would imply that insufficient activation of Notch contributes to the decreased regenerative capacity of aged muscle [33]. Testosterone is an inducer of Notch signaling but plasma testosterone levels are reduced in aged skeletal muscle. This opens the possibility that the impairment of Notch signaling in aged skeletal muscle may be a reduction in testosterone levels [32,33,34]. In human and murine skeletal muscle models, elevated TGFβ levels correlate negatively with decreased Notch signaling in aged skeletal muscle [32]. Furthermore, forced Notch activation inhibits TGFβ-induced upregulation of cyclin-dependent kinase inhibitors (Cdks) in satellite cells. This could indicate the mechanisms underlying the imbalance between TGFβ and Notch affecting regeneration of aged skeletal muscle [33,34].

### 5.2. Wtn Signaling

Dysregulation of Wnt signaling during aging has been proposed to contribute to the loss of Sc function in sarcopenic skeletal muscle [34]. Increased Wnt signaling driven by a ligand of systemic origin promotes conversion of Sc from myogenic to fibrogenic contributing to increased tissue fibrosis and reduced regenerative capacity [35]. Complement protein C1q was identified as the Wnt activator ligand responsible for aging-related phenotypes [24]. In the presence of Wnt inhibitors, Dickoppf (DKK1), or siRNA against β-catenin, a decrease in Wnt signaling occurs which stimulates muscle cell proliferation. Furthermore, in incubation with Frizzled-Fc with serum from aged mice, there was a decrease in Wnt activity, which did not occur in young mice. These data suggest that there is an upregulation of Wnt signaling components in the systemic circulation during aging. Increased Wnt signaling in aged skeletal muscle inhibits myogenic, as Sc under-express the myogenic marker Pax 7 [33,34,35].

Therefore, an imbalance in these Notch/Wnt signaling pathways during the repair of aging skeletal muscle can occur because of attenuation of Notch signaling and exacerbation of Wnt signaling, resulting in dysfunctional repair of aging skeletal muscle

### 5.3. Growth Differentiation Factor 11 (GDF11)

Growth differentiation factor 11 (GDF11), a member of the transforming growth factor β (TGF-β) superfamily of cytokines, is a critical rejuvenation factor in aging cells. Systemic levels of GDF11 have been shown to decrease substantially with age [36]. TGF-β is a family of pleiotropic cytokines with more than 30 members; these cytokines regulate multiple cellular biological procedures such as embryogenesis, homeostasis, and various pathological states, implying a relationship between TGF-β signaling and the onset of age-related diseases [37].

Furthermore, in aged mice treated with recombinant GDF11, Sc frequency and function were observed to increase, along with the number of Sc with intact DNA, and the number of satellite cells with damaged DNA reduced. Treatment with recombinant GDF11 also increased the size and caliber of myofibers and improved the regenerative capacity of Sc. As for the effects on aged muscle, an increase in the size of neuromuscular junctions, a reduction in unusable mitochondria, a reduction in vacuole accumulation, and an improvement in mitochondrial patterning (function, mitophagy, and mitochondrial biogenesis) were observed [38]. Similarly, GDF11 improves outcomes in neurodegenerative and neurovascular diseases [36]. Thus, its broad biological effects may include the reversal of senescence in clinical applications, as well as the ability to reverse age-related pathological changes and regulate muscle regeneration in patients with sarcopenia. The changes in muscle induced by GDF-11 stimulate at the functional level substantial increases in endurance physical function and strength [38]. The “*rejuvenating*” capacity of GDF11 is quite surprising since the regions of active GDF11 are 90% homologous to myostatin (GDF8), which is known to directly inhibit skeletal muscle growth [36].

### 5.4. Myostatin or Growth Differentiation Factor 8 (GDF8)

Myostatin or GDF8 belongs to the family of TGF-β and is a negative regulator of skeletal muscle development and size. A loss of its function leads to muscular hypertrophy. Myostatin can be disrupted by using specific antibodies that compete with its target. This leads to an increase in lean body mass and an improvement in performance. In addition, myostatin maintains the quiescent state of Sc, and it has been shown that inhibiting myostatin activates Sc although it does not directly regulate it, but rather this activation is a consequence of increased myofiber protein synthesis [38]. Thus, GDF8 inhibition is a potential therapy for muscle wasting diseases, some of which are associated with Sc exhaustion such as sarcopenia.

### 5.5. Peroxisome Proliferator-Activated Receptor Gamma Coactivator 1α (PGC-1α)

Peroxisome proliferator-activated receptor gamma coactivator 1α (PGC-1α) is a key regulator of mitochondrial biogenesis and mitochondrial respiration. PGC-1α plays a critical role in the control of metabolism and energy homeostasis at the myocyte level. PGC-1α expression is activated by calcium/calmodulin kinase IV, resulting in increased mitochondrial content and mitochondrial activity [39]. PGC-1α activation results in the triggering of an autoregulatory mechanism that in turn activates the transcription factor myocyte-specific enhancer factor (MEF2) for motor expression. PGC-1α is also involved in the fiber shift toward oxidative type I fibers. Increases in PGC-1α are fiber-specific and may contribute to fiber type distribution changes after endurance training [40]. Overexpression of this biomarker ameliorates defects produced by β-oxidation caused by high lipid load in myocytes. Activation of PGC-1α prevents the onset of myopathy and prolongs mitochondrial lifespan in mice, leading to the suggestion that PGC-1α upregulation may partially compensate for widespread mitochondrial defects. Induction of mitochondrial biogenesis through transgenic expression of PGC-1α in skeletal muscle delayed the onset of myopathy and marked a prolonged lifespan of muscle cells [41]. Therefore PGC-1α plays an important role in aging and related diseases such as sarcopenia. In this regard, PGC-1α can prevent Sc cell apoptosis and markedly increase the myogenic potential of Sc. In addition, PGC-1α triggers Sc niche remodeling by altering extracellular matrix composition, including fibronectin levels, which positively affects the proliferative output of Sc, whose gene expression and protein levels are inversely modulated by PGC-1α [42]. Increasing Sc would potentially have a therapeutic action in sarcopenia in which myogenic capacity is reduced. This has been corroborated in murine models where PGC-1α protects against a variety of muscle wasting conditions including fiber atrophy or dystrophic pathologies [43].

### 5.6. Sirtuin 1 (Sirt1)

Sirtuin 1 (Sirt1), a nicotinamide adenine dinucleotide (NAD+) deacetylase that is activated by caloric restriction, has been identified as an important regulator of skeletal muscle metabolism [44]. Elevated Sirt1 activity has been shown to increase Sc proliferation, and Sirt1 has also been shown to inhibit the differentiation of C2C12 myoblasts (activated murine Sc) and to reduce myogenin expression (a stimulator of Sc differentiation). Sirt1 could delay differentiation and prolong or increase Sc proliferation [45]. PGC1-α biomarker, in addition to its role in Sc metabolism and regulation, deacetylates and activates PGC1-α, which in turn activates factors with anti-inflammatory effects such as PARP-α [41]. Therefore, PGC1-α has a beneficial role in longevity conferring potential properties that counteract the deterioration associated with muscle aging.

### 5.7. Paired Box 7 (Pax 7)

Pax genes play key roles during development. Members of this family of paired box/homeodomain transcription factors regulate the contribution of progenitor cells to different tissue types. Paired box 3 (Pax3) and its paralogue paired box 7 (Pax7) have been implicated in the specification of cells that will enter the myogenic program [46]. Pax7 is a transcription factor involved in the specification and maintenance of Sc. Muscle Sc uniformly express Pax7 in all skeletal muscles of the body. Pax7 is also expressed in the central nervous system [47]. During embryonic development and postnatal growth, Pax7(+) Sc actively contribute to the increase in myonuclei in growing neonatal myofibers. In the adult, Pax7(+) Sc can repair damaged muscle tissue. The sarcopenic process involves a greater number of residual Sc, this subpopulation, which is more compromised for efficient skeletal muscle regeneration functions, expresses significantly lower levels of Pax7, and consequently its self-renewal and survival potential is markedly lower [48]. During aging, the function of Pax7 decreases, and the deposition and maintenance of the skeletal muscle Sc pool is impaired resulting in the overall decrease in the regenerative capacity of old muscle. Administration of diphtheria toxin induces a significant decrease in Pax7 (+) Sc, which is associated with atrophies in muscle differentiation and regeneration [49]. These findings have demonstrated that Pax7(+) cells are muscle Sc and therefore shown to be an essential biomarker to know the Sc status. Reversal of Pax7 levels could provide an opportunity for new therapies against sarcopenia.

### 5.8. Paired Box 3 (Pax 3)

Pax 3 is initially expressed in the presomitic paraxial mesoderm during development and is required for the formation of hypaxial muscles, including limb muscles [50]. Pax3 is required to maintain the migration of Sc in the somite and their migration to myogenic sites. Therefore, Pax3 could play a key role in maintaining skeletal muscle cell proliferation. These properties make Pax3 play an essential role in adult muscle homeostasis and skeletal muscle repair, promoting the contribution of muscle Sc to the balance of muscle structure and/or function, generating a beneficial response to the biological stress that Sc undergo [51]. This agrees with ex vivo studies where Pax3-induced expansion allows for genetic correction of dystrophic Sc. These results demonstrate that Pax3 can promote ex vivo development of Sc while maintaining their stem cell regenerative properties [52]. High levels of Pax3 could induce the expression of MyoD and Myf5 by direct binding and trans-activation of their enhancers in vitro responsible for the myogenic differentiation of Sc [50].

### 5.9. Myogenic Regulatory Factors (MRFs)

MyoD, Myf5, myogenin (MyoG), and MRF4 are myogenic regulatory factors (MRFs). MRFs are transcription factors that modulate the process of myogenesis and repair of injured or aged muscle. In addition, MRFs induce the transformation of Sc into mature muscle lineage cells [53]. Additionally, MRFs modulate myogenesis by controlling the expression of myogenic and non-myogenic genes [54]. Sc are usually quiescent during the cell cycle; however, when these are activated by stimulation, such as by muscle injury, MRF expression increases, and Sc progenitors interact on the myoblast lineage and proliferate rapidly [55]. MRFs sequences showed similarity between: MyoD and Myf5 53%; MyoG and MyoD 38%; MyoG and Myf5 40%; MRF4 and MyoD 43%; MRF4 and Myf5 40%. In addition, MRFs can induce myogenesis and are expressed in some non-myogenic cells, such as fibroblasts, adipose, nerve and liver cells, indicating a functional overlap in the establishment of the muscle cell lineage [56].

Newborn mice without MyoD or Myf5 have been reported to be completely deficient in myoblasts and myofibers [57]. MyoD-/Myf5- Sc are maintained without activity in aged and damaged muscle, which would indicate that the decreased capacity for muscle restoration is not only due to the loss of Sc, but also to the absence of MRFs [55]. Therefore, MyoD and Myf5 are essential for Sc and their transformation into muscle cells and act upstream of MRF4 and MyoG [54]. Asfour et al. [53] reported that Myf5 is responsible for activating all other MRFs (MyoD, Myogenin, and MRF4) and MEF2 proteins.

The transcription factor MyoG regulates myocyte fusion during development, and its function in adult myogenesis is to coordinate Sc activation and return to quiescence [58]. MyoG has skeletal muscle differentiation-inducing effects that cannot be substituted by other myogenic regulatory factors. MyoG participates in the formation of new myotubes, control myocyte fusion, and influences the beneficial Sc to niche ratio by stimulating differentiation and transformation signals from Sc to myocytes [59]. This could demonstrate the multilevel collaboration of MyoG to muscle homeostasis. Loss of MyoG deregulates mTORC1 signaling, generating alterations in early Sc activation and in the Sc cell cycle, concomitant with deregulation of other MRFs (MyoD, Myf5) [54]. MyoG depletion has functional effects because it markedly decreases myofiber generation, induces reduced myotube growth, and Sc are shown to be unable to respond to muscle injury repair stimuli [59]. Thus, MyoG is essential for the development of functional skeletal muscle. Transplantation of Sc overexpressing MyoG (Sc MyoG+) delayed muscle atrophy in a murine model of skeletal muscle denervation. Muscles transplanted with Sc MyoG+ showed significant increases in MyoG expression with muscle mass and muscle fiber cross-sectional area increases [60]. MyoG has an autoregulatory mechanism and can induce terminal differentiation directly or indirectly through MRF4 interaction [53]. These results place MyoG as an essential biomarker for monitoring muscle behavior in sarcopenia. Furthermore, acting on MyoG could establish a new therapy on damaged muscles.

MRF4 is expressed exclusively in skeletal muscle and is expressed at higher levels in adult muscle fibers than all other genes in the MRFs family [61]. MRF4 acts as a negative regulator of muscle growth. The genes that regulate the hypertrophic response of Sc are controlled by MRF4 which inhibits their activation by MEF2, acting as a positive regulator of muscle growth [62]. In an animal model, MRF4+ blocked Sc activity delaying muscle fiber regeneration [63]. Thus, the MRF4-MEF2 axis controls muscle growth and opens a new perspective to prevent muscle involution in sarcopenia.

### 5.10. CD34

CD34, a transmembrane-localized protein of the sialomucin family, is an accepted and clinically exploited marker of adult hematopoietic stem cells and early blood cell progenitors [64]. CD34 is also present on quiescent Sc in adult skeletal muscle, which may suggest that CD34 plays a critical role in adult muscle tissue regeneration. In this sense, CD34 (truncated) is expressed on quiescent Sc and that activation is accompanied by a transient switch to full CD34 expression on active Sc. The two CD34 isoforms could have distinct functions in maintaining activation during muscle tissue renewal and regeneration [65]. In this regard, it has been reported that CD34 is necessary for efficient muscle regeneration in response to acute and chronic damage. The downregulation of CD34 expression levels in myogenic precursors following Sc CD34-graft-associated muscle tissue injury in an animal model would indicate the direct involvement of CD34 in adult myogenesis [66]. Overall, CD34 would be a useful biomarker of Sc, and CD34 is required for the progression of Sc to efficient muscle tissue in response to acute and chronic damage.

### 5.11. p16ink4a: P16 Cyclin-Dependent Kinase 2A Inhibitor

p16ink4a is a cyclin-dependent kinase 2A inhibitor widely expressed in muscle. p16ink4a is a negative regulator of normal cell proliferation and its expression is significantly increased in aging, limiting proliferation and renewal of Sc [67]. Increased expression of p16INK4a with age contributes to the decreased regenerative capacity of pancreatic islets, decreased forebrain progenitors and neurogenesis, and finally, defects in hematopoietic stem cell repopulation [68]. With respect to muscle, it has been found in the skeletal muscle of the BubR1 mouse, in which p16INK4a contributes to the acquisition of age-related pathologies; however, suppression of p16INK4a could attenuate the progression of these pathologies when they are already established, including sarcopenia [69].

## 6. Musculoskeletal Tissue Biopsy Samples Are a Key Diagnostic Tool

Skeletal muscle biopsy remains an important investigative tool in the diagnosis of a variety of muscle disorders including sarcopenia. Muscle weakness, myopathic changes, and increased plasma creatine kinase (CK) that are caused by sarcopenia would be clinical indicators for muscle biopsy [70]. The target muscles to be biopsied are weak muscles that can overcome gravity. Very weak muscles show a marked loss of muscle fibers with the replacement of adipose or connective tissue [7]. A closed biopsy is probably the most appropriate for determining muscle status in older adults with sarcopenia because it is minimally invasive and potentially yields more than one sample [70]. However, it is not without drawbacks because it is not easy to obtain from some muscles, it is expensive, it has a long processing time, it is impossible to tolerate throughout the aging process, the assessment of the heterogeneity of sarcopenia is limited to that of the biopsy analyzed, and it is not easy to follow the response to interventions in the elderly [28].

### Characterized the Human Muscle Proteome

Different methodologies (direct sample lysis; two-dimensional peptide separation; differential solubilization; one-dimensional SDS-PAGE gel electrophoresis; tandem mass spectrometry with high-performance electrospray ionization) have been used to identify and quantify the totality of proteins that make up muscle tissue [71,72]. In fact, more than 2000 proteins have been recognized that make up skeletal muscle in healthy subjects [71]. The most abundant proteins are derived from the sarcomeres and cytoplasm of multinucleated muscle cells, as well as from non-muscle tissue, such as red blood cells, inflammatory cells, and connective tissue [71]. In addition to many contractile protein isoforms, all proteins involved in major glucose and lipid metabolic pathways in skeletal muscle were detected. Mitochondrial proteins accounted for 22% of the total proteins identified. In addition, 55 protein subunits belonging to respiratory complex IV were distinguished. Enzymes involved in metabolic and endocrine signaling pathways as well as in calcium homeostasis were also recognized [72]. From the characterization of the proteome of healthy subjects, quantitative changes in the muscle proteome of patients affected by disorders affecting skeletal muscle could be evaluated.

## 7. Role of the Proteome in Aging

Aging is a social and biological concept whose definition and perception have evolved throughout history. Nowadays, individuals enjoy greater longevity, although this is not always accompanied by good health or quality of life. The mechanisms linked to aging identify nine key points of cellular alteration that mediate this process: increased senescence-associated secretory phenotype (SASP), stem cell depletion, altered cell communication, genomic instability, loss of telomere material, functional alteration in mitochondria, impaired nutrient perception, loss of proteostasis, and epigenetic alterations [73]. Proteomics allows the comprehensive cataloging of complete protein complements as a tool to identify protein alterations in aging. Proteome analysis gives us a complete approach in heterogeneous and plastic tissues, such as skeletal muscle of the whole protein complement [74]. The elderly have fewer copies of ribosome-forming proteins altering the synthesis of new proteins, and fewer proteins are involved in cellular energy metabolism producing energy inefficiently. In addition, increased protein destruction triggers an elevated production of inflammatory molecules. Therefore, aging would cause an alteration of proteostasis homeostasis, deregulating the dynamics of a balanced functional proteome [75]. One of the characteristics of aging on muscle tissue is the accumulation of oxidatively modified proteins, decreasing the quality of the cellular proteome. Oxidized proteins, ‘oxy-proteome’, are damaged proteins that accumulate and disrupt key cellular functions such as cell morphology and transport, muscle contraction, and energy metabolism [76]. Additionally, the information provided by proteomic analysis has provided insight into an age-related shift towards slower protein isoforms of myosin heavy chain, myosin light chain, actin, and tropomyosin. Proteomic profiling has also revealed an increase in mitochondrial enzymes with a decrease in glycolytic enzymes during the fast-to-slow transformation process in aged skeletal muscle [77]. That is, proteomic profiling of aged muscle tissues has shown changes in metabolic markers that are characteristic of a fast-to-slow transition process (several glycolytic enzymes, such as pyruvate kinase and numerous mitochondrial enzymes), as well as changes in adenylate kinase and several molecular chaperones [71]. Additionally, Ubaida-Mohien et al. [78] showed that RNA in the elderly had more alternative transcripts, so changes in protein levels modified muscle viability and functionality.

Some sarcopenic patients, in advanced stages, suffer mild or moderate stages of cachexia. The use of muscle biopsy has provided insight into a cachexia-specific protein signature. This is characterized by changes in muscle contractile myosin and energy metabolism. That is, mitochondrial dysfunction and alterations in contractile fibers are the changes in the proteome that would identify patients with sarcopenia-derived cachexia [79].

### Changes in the Proteome after Physical Exercise

Physical exercise is one of the most widely used measures to delay/modulate the evolution of sarcopenia [19]. In this sense, proteomic analysis was performed on human muscle biopsies by mass-spectrometry-based proteomic technique before and after the practice of physical exercise. This analysis made it possible to identify changes in the expression, after physical exercise, of 237 slow contraction proteins and 172 fast contraction proteins, in skeletal muscle fibers. The alterations in expression levels were mainly in secreted proteins and proteins involved in the transcription of mitochondrial metabolism, Ca^2+^ signaling, fat metabolism, and glucose metabolism [80]. All these proteins undergo beneficial adaptations after physical training that modulate changes in aging.

## 8. Potential Proteomic Biomarkers Involved in Aging

Proteins are particularly attractive because they are direct biological effectors and dynamically change with aging [74]. In this sense, protein identification could prove to be a very promising tool as a potential biomarker in sarcopenia.

### 8.1. Growth Differentiation Factor 15 (GDF-15)

The study of proteomes identifies a potential link between post-translational modification (PTM) and metabolism in long-lived mammals [73]. For this purpose, the OMAscan test, which uses single-stranded nucleic acids or oligonucleotides, aptamers, present in proteomes, is a key tool to identify proteins related to various cellular mechanisms of aging [81]. In addition, it would allow the identification of several proteins that show variability in their serum values in relation to the chronological age of the individual [82].

Tanaka et al. [82] studied a population of 240 men and women aged 22–93 years, with no record of pathologies, medical treatments, or cognitive alterations. They measured 1301 plasma proteins, of which 197 proteins were positively associated and 20 proteins were not associated with the age of the individuals. These investigators [71] demonstrated a strong association between age and plasma values of growth differentiation factor 15 (GDF-15). GDF-15 is a cytokine secreted mainly by Sc, macrophages, and cardiomyocytes upon oxidative stress or inflammatory processes [83].

In this sense, Lehallier et al. [84] studied a population of 4331 individuals, aged 18–95 years, where 2952 plasma proteins were measured. The results showed changes in the proteomes in the form of peaks at 34, 60, and 78 years of age, which could be indicative of the activation and relationship of different metabolic pathways or proteomes throughout aging. GDF15 was present within the most abundant proteins in all three peaks, including chordin-like protein 1 (CHRDL1) and pleiotrophin growth factor (PTN), with a higher presence at 60 and 78 years. Other data of interest is the presence of changes in certain metabolic pathways in these peaks, such as the presence of a decrease in the regulation of structural proteins of the extracellular matrix at the age of 34 years, which then with the passage of time presents a regression, increased hormonal, and hemodynamic activity at age 60 years and at 78 years an increase in bone morphogenic protein markers [84]. These investigators [84] posit a probable relationship between sex and proteomes, presenting changes in follicle stimulating hormone (FSHB), human chorionic gonadotropin (CGB), prostate antigen (KLK3), sclerostin (SOST), ADP-ribosylation factor protein 2 (ARFIP2), and growth differentiation factor 15 (GDF15) levels. This could indicate that aging is not a linear process, but also that men and women age differently.

Perhaps, both studies [82,84] would allow us to assess aging as a dynamic, heterogeneous, and very broad process. Knowing the linked proteomes and the metabolisms involved in aging allows us to study the possibility of interventions that could slow down, stop, or reverse aging, guaranteeing not only more years of life, but also the quality and healthy years of life.

### 8.2. Protein Alterations in Skeletal Muscle Atrophy

Muscle atrophy is a consequence of aging. Fifteen proteins were oversecreted during muscle atrophy and 11 were under secreted. As for the variation in extracellular matrix proteins, perlecan, fibrillin 1, and biglycan were downregulated, contrary to periostin and collagen IV(α2). As for cytokines and growth factors, only macrophage colony-stimulating factor 1 was downregulated [85]. Alcohol dehydrogenase (NADP (+)), chymotrypsinogen B, and protein disulfide isomerase A3 are 3 enzymes that were found to be more secreted by atrophied skeletal muscle cells. Delta-aminolevulinic acid dehydratase, Obg-type ATPase 1 (hOLA1), and peroxidase homolog were found to be secreted less [80]. Tissue inhibitor of metalloproteinases 2 (TIMP2) was less secreted and serpin family A member 3 (serpin A3n) was more secreted by atrophied skeletal muscle cells. Serpin A3n localizes around the Sc and/or myofibrils and is an extracellular inhibitor of proteases such as granzyme B, trypsin, chymotrypsin, cathepsins G/B/L, and leukocyte elastase [13]. Additionally, follistatin is another protein that is expressed more during musculoskeletal atrophy [85,86].

## 9. Conclusions

We identified a total of 13 potential biomarkers of Sp by their physiological and biological interaction with Sc (Table 1). We report that expression of GDF11, PGC-1α, Sirt1, Pax7, Pax3, Myf5, MyoD, CD34, MyoG, and activation of Notch signaling stimulate the number and activity of Sc which could modulate and delay Sp progression. However, detection in biological fluids of GDF8, p16INK4a, Mrf4, and activation of the Wnt pathway would contribute to early Sp development by directly inducing reduced and/or altered Sc function, which would attenuate the restorative capacity of skeletal muscle. Although no biomarker has been validated, so far, this manuscript could stimulate the application of these biomarkers that could be evaluated by molecular biology techniques linked to liquid biopsy in the clinical management of Sp. Additionally, tissue biopsy remains an important diagnostic tool. Proteomic profiling of aged muscle tissues has shown shifts toward protein isoforms characteristic of a fast-to-slow transition process and an elevated number of oxidized proteins. In addition, a strong association between age and plasma values of GDF-15 has been described and serpin A3n was more secreted by atrophied muscle cells. The identification of these new biomarkers has the potential to change the path of personalized medicine, as it could increase the knowledge of Sp disease, and predict in real time the course of Sp by monitoring its evolution and evaluating responses to possible therapeutic strategies.

## Figures and Tables

**Table 1:** Interaction and modulation of biomarkers with muscle satellite cells.

Biomarker	Physiological and Biological Interaction with Sc		Modulation on Sc
***Notch signaling***	Master regulator of Sc function and its balance controls Sc self-renewal and myogenic differentiation in a coordinated manner		+
***Wtn signaling***	Dysregulation of Wnt signaling during aging has been proposed to contribute to the loss of Sc function in sarcopenic skeletal muscle		−
***Growth differentiation factor 11 (GDF11)***	Sc frequency and function were observed to increase, along with the number of Sc with intact DNA, and the number of Sc with damaged DNA was reduced		+
***Growth differentiation factor 8 (GDF8) or*** ***Myostatin***	Negative regulator of skeletal muscle development and size and Sc		−
***Peroxisome proliferator-activated receptor*** ***gamma coactivator 1α (PGC-1α)***	Prevent Sc cell apoptosis and markedly increase the myogenic potential of skeletal muscle Sc		+
***Sirtuin 1 (Sirt1)***	Increases Sc proliferation, inhibits myoblast differentiation, and reduces myogenin expression		+
***Paired box 7 (Pax 7)***	Sc Pax7(+) repair damaged muscle tissue		+
***Paired box 3 (Pax 3)***	Play an essential role in adult muscle homeostasis and skeletal muscle repair, promoting the contribution of muscle Sc to the balance of muscle structure and/or function		+
***Myogenic regulatory factors (MRFs)***	MyoD and Myf5	Transformation of Sc into muscle cells	+
	Myogenin (MyoG)	Beneficial relationship between Sc and niche by stimulating differentiation signals and transformation of Sc to myocytes	+
	MRF4	Block Sc activity by delaying the regeneration of muscle fibers	−
***CD34***	Necessary for the progression of Sc to efficient muscle tissue in response to acute and chronic damage		+
***P16 cyclin-dependent kinase 2A inhibitor (p16ink4a)***	Limits the proliferation and renewal of Sc		−
***Growth differentiation factor 15 (GDF-15)***	A strong association between age and plasma values		−
Serpin Family A Member 3 (serpin A3n)	Increased secretion by atrophied muscle cells		−

Abbreviations: Sc, muscle satellite cells; +, positive effect; −, negative effect; DNA, deoxyribonucleic acid.; CD34, a cluster of differentiation 34.
